# Local, multimodal intralesional therapy renders distant brain metastases susceptible to PD-L1 blockade in a preclinical model of triple-negative breast cancer

**DOI:** 10.1038/s41598-021-01455-4

**Published:** 2021-11-09

**Authors:** Toshihiro Yokoi, Takaaki Oba, Ryutaro Kajihara, Scott I. Abrams, Fumito Ito

**Affiliations:** 1 Center for Immunotherapy, Roswell Park Comprehensive Cancer Center, Elm and Carlton Streets, Buffalo, NY 14263 USA; 2 Department of Neurosurgery, Shiga University of Medical Science, Otsu, Japan; 3 Division of Breast and Endocrine Surgery, Department of Surgery, Shinshu University School of Medicine, Matsumoto, Japan; 4 Department of Immunology, Roswell Park Comprehensive Cancer Center, Buffalo, NY USA; 5 Department of Surgical Oncology, Roswell Park Comprehensive Cancer Center, Buffalo, NY USA; 6 Department of Surgery, University at Buffalo Jacobs School of Medicine and Biomedical Sciences, The State University of New York, Buffalo, NY USA

**Keywords:** Immunotherapy, Tumour immunology, Vaccines

## Abstract

Despite recent progress in therapeutic strategies, prognosis of metastatic triple-negative breast cancer (TNBC) remains dismal. Evidence suggests that the induction and activation of tumor-residing conventional type-1 dendritic cells (cDC1s) is critical for the generation of CD8^+^ T cells that mediate the regression of mammary tumors and potentiate anti-PD-1/PD-L1 therapeutic efficacy. However, it remains unknown whether this strategy is effective against metastatic TNBC, which is poorly responsive to immunotherapy. Here, using a mouse model of TNBC, we established orthotopic mammary tumors and brain metastases, and treated mammary tumors with in situ immunomodulation (ISIM) consisting of intratumoral injections of Flt3L to mobilize cDC1s, local irradiation to induce immunogenic tumor cell death, and TLR3/CD40 stimulation to activate cDC1s. ISIM treatment of the mammary tumor increased circulating T cells with effector phenotypes, and infiltration of CD8^+^ T cells into the metastatic brain lesions, resulting in delayed progression of brain metastases and improved survival. Furthermore, although anti-PD-L1 therapy alone was ineffective against brain metastases, ISIM overcame resistance to anti-PD-L1 therapy, which rendered these tumor-bearing mice responsive to anti-PD-L1 therapy and further improved survival. Collectively, these results illustrate the therapeutic potential of multimodal intralesional therapy for patients with unresectable and metastatic TNBC.

## Introduction

Triple-negative breast cancer (TNBC), characterized by the lack of estrogen receptor (ER), progesterone receptor (PR), and human epidermal growth factor receptor 2 (HER2/neu), is an aggressive subtype of breast cancer with limited treatment options^1^. Women with TNBC have a higher rate of early distant relapse compared to those with other subtypes of breast cancer, and thus is associated with poor clinical outcomes despite having an initial good response to chemotherapy^1^. Compared to other types of breast cancer, TNBC is more likely to metastasize to visceral sites, particularly to the lungs and brain^1^. Prognosis of patients with TNBC brain metastases is almost uniformly poor, with a median survival of 4.9 months after the development of brain metastases^2,3^. Therefore, new treatment strategies for TNBC brain metastases are an unmet clinical need.

Notably, TNBC exhibits a higher level of PD-L1 expression associated with the presence of tumor-infiltrating lymphocytes (TILs), and a higher degree of mutational burden compared with other subtypes of breast cancer^4–8^, suggesting that TNBC might be more immunogenic, and thus can be an attractive target of immunotherapy such as PD-1/PD-L1 blockade therapy. Indeed, the efficacy of anti-PD-L1 blockade (atezolizumab) combined with nab-paclitaxel has recently been demonstrated in TNBC in the advanced or metastatic setting^9^; progression-free survival was significantly longer in the atezolizumab–nab-paclitaxel group than in the placebo–nab-paclitaxel group (median, 7.2 months vs. 5.5 months; 95% confidence interval 0.69 to 0.92; P = 0.002). However, given that durable responses in patients treated with anti-PD-1/PD-L1 therapy and chemotherapy are rare, there is both scientific and clinical justification to explore novel therapeutic strategies to further improve patient outcomes^9^.

The tumor immune microenvironment (TME) plays a key role in clinical outcomes of patients with many cancer types, including TNBC. The frequency of TILs substantially influences the likelihood of achieving a pathologic complete response (pCR) to neoadjuvant chemotherapy and improve patient prognosis^10,11^. Furthermore, the presence of TILs in TNBC also correlates with response to immunotherapy, which is associated with a high level of PD-L1 expression^12,13^. Therefore, increasing CD8^+^ TILs are likely to improve response to immunotherapy and/or chemo-immunotherapy.

Dendritic cells (DCs) are a diverse group of specialized antigen-presenting cells (APCs) that play critical roles in linking innate and adaptive immunity^14,15^. Among various subsets of DCs, Batf3-dependent conventional type 1 dendritic cells (cDC1s; migratory CD103^+^ and lymphoid CD8α^+^ DCs in mice, and CD141^+^ DCs in humans) have enhanced abilities to phagocytose dead cells and transport tumor-associated antigens (TAAs) to tumor-draining lymph nodes (TdLN), where they cross-present TAAs to CD8^+^ T cells^16^. Evidence revealed that cDC1s in the TME play pivotal roles in the priming and expansion of tumor-specific CD8^+^ T cells^17–22^, promoting their infiltration into the TME^23^, and thus enhancing the efficacy of anti-PD-1 therapy^24–26^. Although cDC1s are rare populations in the TME, they can be induced by in situ or systemic administration of Fms-like tyrosine kinase 3 receptor ligand (Flt3L)^17,18,24–29^.

In situ immunomodulation (ISIM) is a multimodal intralesional therapy comprised of in situ delivery of Flt3L, radiotherapy, and dual CD40/TLR3 stimulation^29^. Using multiple orthotopic murine tumor models of poorly T cell-inflamed tumors including TNBC, we have demonstrated that ISIM: mobilizes cDC1s to the TME; induces maturation of cDC1s; facilitates trafficking of cDC1 carrying TAAs to the TdLN; elicits de novo adaptive T cell immunity; triggers rapid regression of primary tumors, as well as non-irradiated contralateral tumors; renders non-T cell-inflamed tumors responsive to anti-PD-L1 therapy; reshapes clonally expanding T-cell receptor (TCR) repertoires in tumors; overcomes acquired resistance to anti-PD-L1 therapy, resulting in eradication of tumors; and develops tumor-specific systemic immunological memory^29,30^. However, whether ISIM develops enhanced systemic antitumor immunity and controls distant brain metastases remains unknown, which has significant translational implications for those patients with few, if any, therapeutic options.

In this study, using an orthotopic mouse model of TNBC brain metastases, we hypothesized that ISIM-induced local immunity led to systemic antitumor activity that delayed the progression of established brain metastases and improved survival, alone or in combination with anti-PD-L1 therapy. The results from this study support our central hypothesis and reveal the translational potential of ISIM for patients with unresectable or metastatic TNBC.

## Methods

### Mice

Female C57BL/6 mice were purchased from the Jackson Laboratories. All mice were age matched of 7–9 weeks old at the beginning of each experiment. Mice were maintained under specific pathogen-free conditions and housed in the Laboratory Animal Resources facility. All studies were conducted in accordance with ARRIVE guidelines and approved by the Institutional Animal Care and Use Committee (IACUC) at the Roswell Park Comprehensive Cancer Center.

### Cell lines

The AT-3 tumor cell line was established from a primary mammary gland carcinoma of the PyMT-MMTV transgenic mice on a B6 strain and was maintained as described^31^. AT-3 cells were cultured in Gibco Dulbecco’s Modified Eagle Medium supplemented with 10% fetal bovine serum (FBS) (Sigma), 1% non-essential amino acid (NEAA) (Gibco), 2 mM l-glutamine (Gibco), 0.5% penicillin/streptomycin (Gibco), and 55 μM 2-mercaptoethanol (Gibco). AT-3 tumor cells expressing luciferase (AT-3-luc) were generated with infection of lentiviruses encoding luciferase (pLenti PGK V5-LUC Neo, Addgene plasmid #21471). These cell lines were authenticated by morphology, phenotype, and growth, and routinely screened for *Mycoplasma*, and were maintained at 37 °C in a humidified 7% CO2 atmosphere.

### Tumor inoculation

AT-3 (5 × 10^5^) tumor cells were orthotopically implanted into the fourth mammary gland of female mice under anesthesia with isoflurane. Brain metastases were established by intra-cardiac injection of AT-3-luc (1 × 10^6^) tumor cells through the fourth intra-costal space under anesthesia with isoflurane as previously described^32,33^.

### In situ immunomodulation (ISIM)

Tumor-bearing mice were treated with hFlt3L (10 μg/dose; Celldex Therapeutics, Inc.) in 30 μL PBS or control PBS intratumorally daily for 5 days. Local irradiation of orthotopic mammary tumors was described^29^. In brief, the mice were anesthetized with isoflurane and positioned under a 2 mm thick lead shield with small apertures limiting exposure to the tumors. The tumor received 9 Gy local irradiation with an orthovoltage X-ray machine (Philips RT250, Philips Medical Systems) at 200 kV using a 1 × 2 cm cone. One day after radiotherapy, mice were treated with injection of high molecular weight poly(I:C) (50 μg/dose; InvivoGen) and agonistic anti-CD40 Ab (50 μg/dose; clone FGK4.5, BioXcell) at the peritumoral site subcutaneously. For the second and subsequent ISIM treatments in a serial ISIM protocol, PBS or Flt3L was injected starting 1 day after TLR3/CD40 stimulation for 5 days. Radiotherapy and TLR3/CD40 agonists were given 1 and 2 days after completion of Flt3L or PBS injection, respectively. Mammary tumor growth was measured 3–4 times a week, and the volumes were calculated by determining the length of short (l) and long (L) diameters (volume = *l*^2^ × *L*/2). Experimental endpoints were reached when mice became moribund and showed neurological focal signs of cachexia, lateral recumbency, lack of response to noxious stimuli.

### Anti-PD-L1 therapy

Anti-PD-L1 antibody (Ab) (clone 10F.9G2, BioXcell) or isotype rat IgG2b (clone LTF-2, BioXcell) was injected intraperitoneally (*i.p.*) every third day at a dose of 200 μg/mouse^29,30,34–36^ starting on the day radiotherapy was performed.

### In vivo bioluminescence imaging

To monitor brain metastases of established AT-3-luc tumors, mice were injected with d-luciferin (1.5 mg/20 g body weight) intraperitoneally, and images were obtained by in vivo bioluminescence imaging (IVIS Spectrum imager) was used as described^29^.

### Flow cytometry

Single cell suspensions of spleens were blocked with anti-mouse CD16/32 (BioLegend) and surface stained with the indicated markers, and evaluated by flow cytometric analysis as described^18,29,35–37^. The following Abs were used; anti-CD45 (clone 30-F11, Invitrogen), anti-PD-1 (clone 29F.1A12, BioLegend), anti-CX3CR1 (clone SA011F11, BioLegend), anti-KLRG1 (clone 2F1/KLRG1, BioLegend), anti-CD4 (clone GK1.5, BioLegend), anti-CD8α (clone 53-6.7, BD Biosciences), and anti-CD3 (clone 145-2C11, BioLegend). LIVE/DEAD Fixable Near-IR Dead Cell Stain kit (Thermo Fisher Scientific)-stained cells were excluded from the analysis. Samples were acquired by Fortessa (BD Biosciences) cytometers, and analyzed with FlowJo software (Treestar).

### Immunohistochemistry staining

The frequency of Immunohistochemistry (IHC) staining was performed as described^29^. To identify CD8^+^ cells in brain, anti-CD8α (1:400, Clone D4W2Z, Cell Signaling Technology) was used. Images were obtained with a Zeiss Axio Imager Z1. The number of cells showing CD8 positivity in each individual high-power field (HPF) (× 200) was quantified across five fields or more of nonsequential cryosections 9 mm in thickness.

### Statistical analysis

Statistical analysis was performed using two-tailed Student’s *t*-test for comparisons between two groups, one-way ANOVA with Tukey’s multiple comparisons for comparisons of more than two groups, or the Mantel-Cox method (log-rank test) for survival analysis using GraphPad Prism 8.02 (GraphPad Software)^29,30^. *P* < 0.05 was considered statistically significant.

### Ethics approval

All animal studies were reviewed and approved by the Roswell Park institutional animal care and use program and facilities (protocol #1316M and 1356M). All aspects of animal research and husbandry were conducted in accordance with the federal Animal Welfare Act and the NIH Guide for the Care and Use of Laboratory Animals. Further, all methods are reported in accordance with ARRIVE guidelines (https://arriveguidelines.org).

## Results

### In situ immunomodulation (ISIM) of mammary tumors with Flt3L, radiotherapy and TLR3/CD40 agonists controls established brain metastases and improves survival

To test systemic antitumor efficacy of ISIM against brain metastatic TNBC, we utilized an established brain metastasis model by intra-cardiac injection of AT-3-luc tumors^32^.

**Figure 1:**
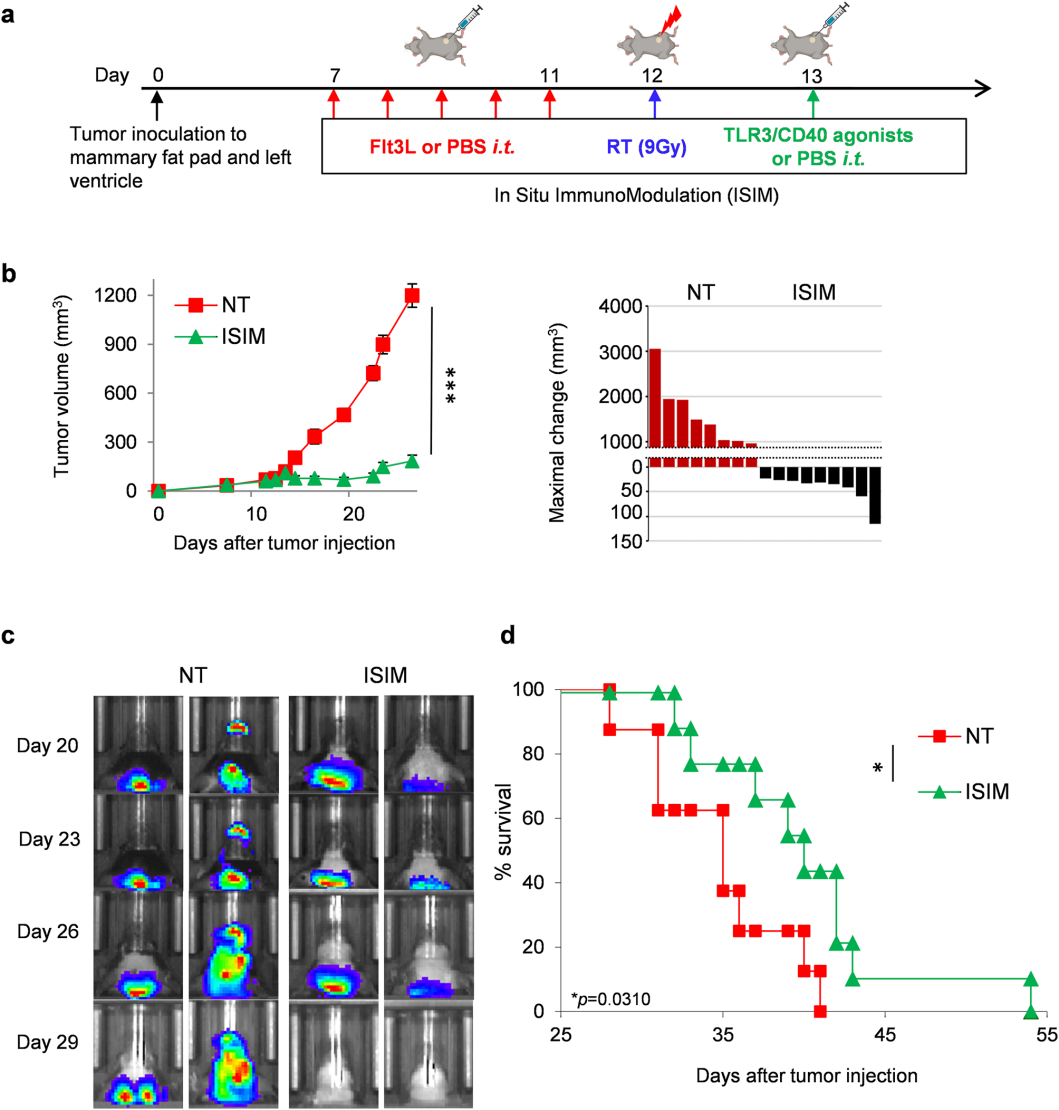
ISIM treatment of mammary tumors with Flt3L, radiotherapy and TLR3/CD40 agonists controls established brain metastases and improves survival. (**a**) Treatment protocol of ISIM in mice bearing established orthotopic mammary and brain metastatic tumors. Illustrations here were created with www.Biorender.com. (**b**) Tumor volume curves (mean) and waterfall plots of AT-3 mammary tumors in mice bearing AT-3-luc brain metastatic tumors. Orthotopic mammary tumors were treated with PBS (NT) or ISIM. Waterfall plots show maximal change of tumor volume at the day when TLR3/CD40 agonists were administered. (n = 8–9 per group) Hazard Ratio (HR) 3.728, confidential interval (CI) 1.128–12.32. (**c,d**) Representative bioluminescent imaging (**c**) of AT-3-luc brain metastases and survival curves (**d**) in mice receiving different treatments against mammary AT-3 tumors, as indicated. (n = 8–9 per group). *P < 0.05, ***P < 0.001, two-tailed unpaired Student *t*-test (**b**), or log-rank (Mantel-Cox) test (**d**). Mean ± SEM. Data shown are representative of two independent experiments.

AT-3 tumor cells were orthotopically implanted into the fourth mammary gland, and AT-3-luc tumor cells were injected into the left ventricle under anesthesia (Supplementary Fig. S1a). Establishment and progression of brain metastases were confirmed and monitored by IVIS. (Supplementary Fig. S1b). Mammary tumors were treated with ISIM comprised of intratumoral injections of Flt3L to recruit cDC1s into the TME, radiotherapy to induce immunogenic tumor cell death and maturation of DCs, and in situ administration of TLR3/CD40 agonists to facilitate trafficking of Ag-loaded cDC1s to TdLN and the subsequent priming and expansion of tumor-specific CD8^+^ T cells^29^ (Fig. 1a). Control mice received intratumoral injections of PBS. ISIM mediated effective regression of orthotopic AT-3 mammary tumors with substantially delayed tumor growth (Fig. 1b). Untreated mice showed progressive growth of brain metastases (Fig. 1c). In contrast, ISIM treatment of orthotopic mammary tumors controlled the growth of distant brain metastases and substantially improved survival (Fig. 1d).

### ISIM facilitates activation and effector differentiation of CD4^+^ and CD8^+^ T cells

**Figure 2:**
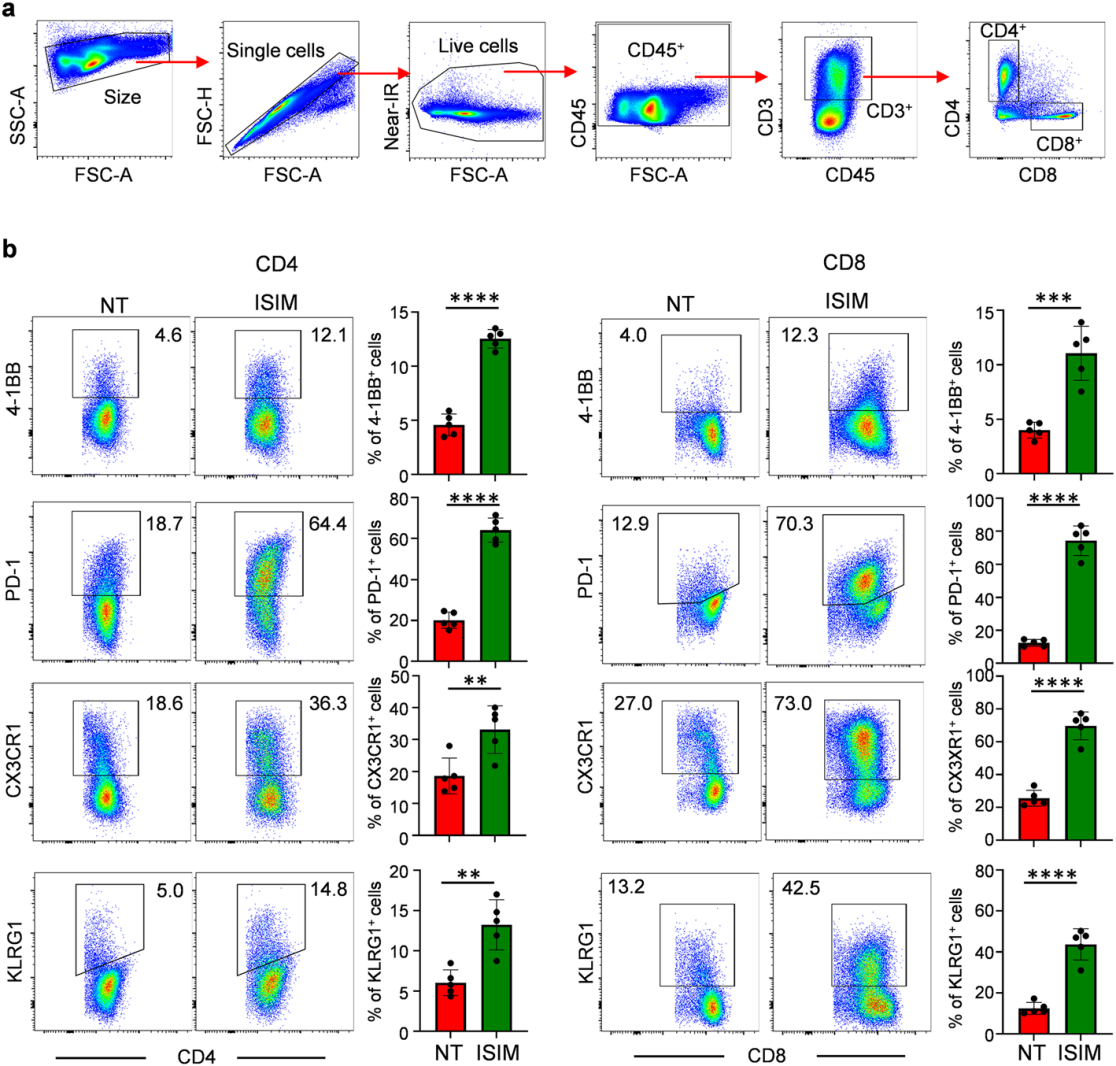
ISIM facilitates activation and effector differentiation of CD4^+^ and CD8^+^ T cells. (**a**,**b**) AT-3 and AT-3-luc tumor cells were inoculated into the fourth mammary gland and left ventricle, respectively. Orthotopic mammary tumors were treated with PBS (NT) or ISIM and spleens were collected 7 days after intratumoral administration of TLR3/CD40 agonists. (**a**) Gating strategy for phenotypic analysis of CD4^+^ and CD8^+^ T cells in the spleen. (**b**) Representative flow cytometric plots showing 4-1BB, PD-1, CX3CR1 and KLRG1 expression in CD4^+^ T cells (left) and CD8^+^ T cells (right) in spleen. Numbers, percent positive cells. Data panels show mean percentage of positive cells in CD4^+^ and CD8^+^ T cells (*n* = 5 per group). **P < 0.01, ***P < 0.001, ****P < 0.0001, two-tailed unpaired Student *t*-test. Mean ± SD. Data shown are representative of two independent experiments.

To gain insights into the potential systemic antitumor efficacy of ISIM treatment of mice bearing orthotopic mammary tumors with brain metastasis, we evaluated the phenotype of CD4^+^ and CD8^+^ T cells in the spleens of untreated or ISIM-treated mice by flow cytometry (Fig. 2a). Markedly increased frequencies of CD4^+^ and CD8^+^ T cells expressing markers of activation (PD-1, 4-1BB), differentiation (CX3CR1), and effector phenotype (KLRG1) was observed in ISIM-treated mice compared to untreated mice (Fig. 2b), suggesting effective activation of adaptive T cell immunity in mice bearing orthotopic mammary tumors with brain metastatic disease in response to ISIM.

### ISIM promotes CD8^+^ T cell infiltration into intracranial lesions

**Figure 3:**
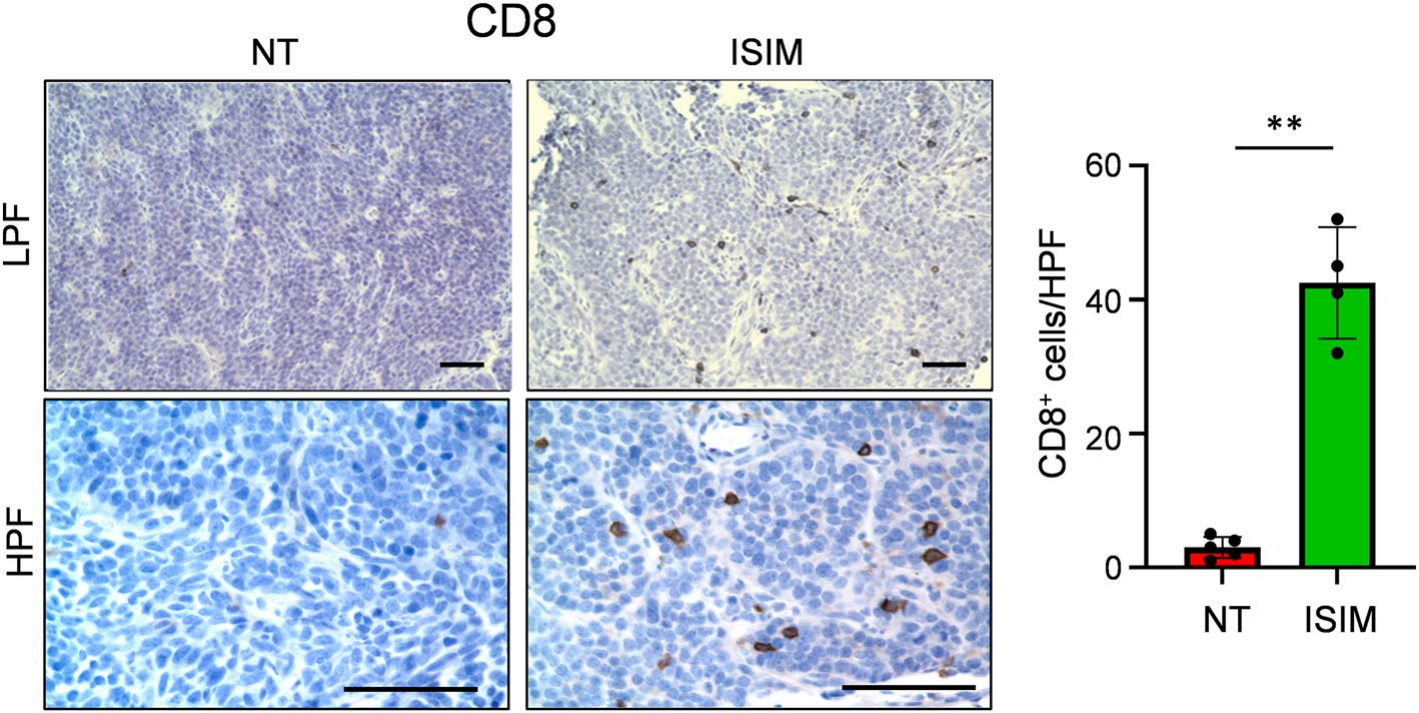
ISIM promotes CD8^+^ T cell infiltration into intracranial lesions. AT-3 and AT-3-luc tumor cells were inoculated into the fourth mammary gland and left ventricle, respectively. Orthotopic mammary tumors were treated with PBS (NT) or ISIM, and brain tumor tissues were collected 7 days after intratumoral administration of TLR3/CD40 agonists. Representative images of immunohistochemistry for CD8^+^ T cells in brain tumor tissue. Images in low-power field (LPF) (upper) and high-power field (HPF) (lower) are shown. Scale bars, 100 µm. A data panel shows mean numbers of CD8^+^ T cells per each HPF within 5 different areas for each tumor (*n* = 4–5 per group). **P < 0.01, two-tailed unpaired Student *t*-test. Mean ± SD. Data shown are representative of two independent experiments.

Next, we investigated whether ISIM treatment of mammary tumors could facilitate the infiltration of CD8^+^ T cells into the distant brain metastases. To this end, we treated mice bearing orthotopic mammary tumors and established brain metastases with ISIM and collected brain tissue 7 days after completion of the regimen. Immunohistochemical (IHC) analysis revealed sparse CD8^+^ T cells in intracranial metastatic lesions in untreated mice (Fig. 3). In contrast, the frequency of CD8^+^ T cells in brain metastases was substantially increased in ISIM-treated mice compared to untreated mice (Fig. 3). These results suggest that ISIM treatment of primary tumors triggers systemic antitumor immunity and generates CD8^+^ T cells that could pass through the blood brain barrier, and traffic to metastatic sites, causing immune remodeling and regression of untreated intracranial brain tumors.

### Serial ISIM treatment of mammary tumors and anti-PD-L1 therapy exhibit synergistic antitumor efficacy and improves survival in mice with established brain metastases

**Figure 4:**
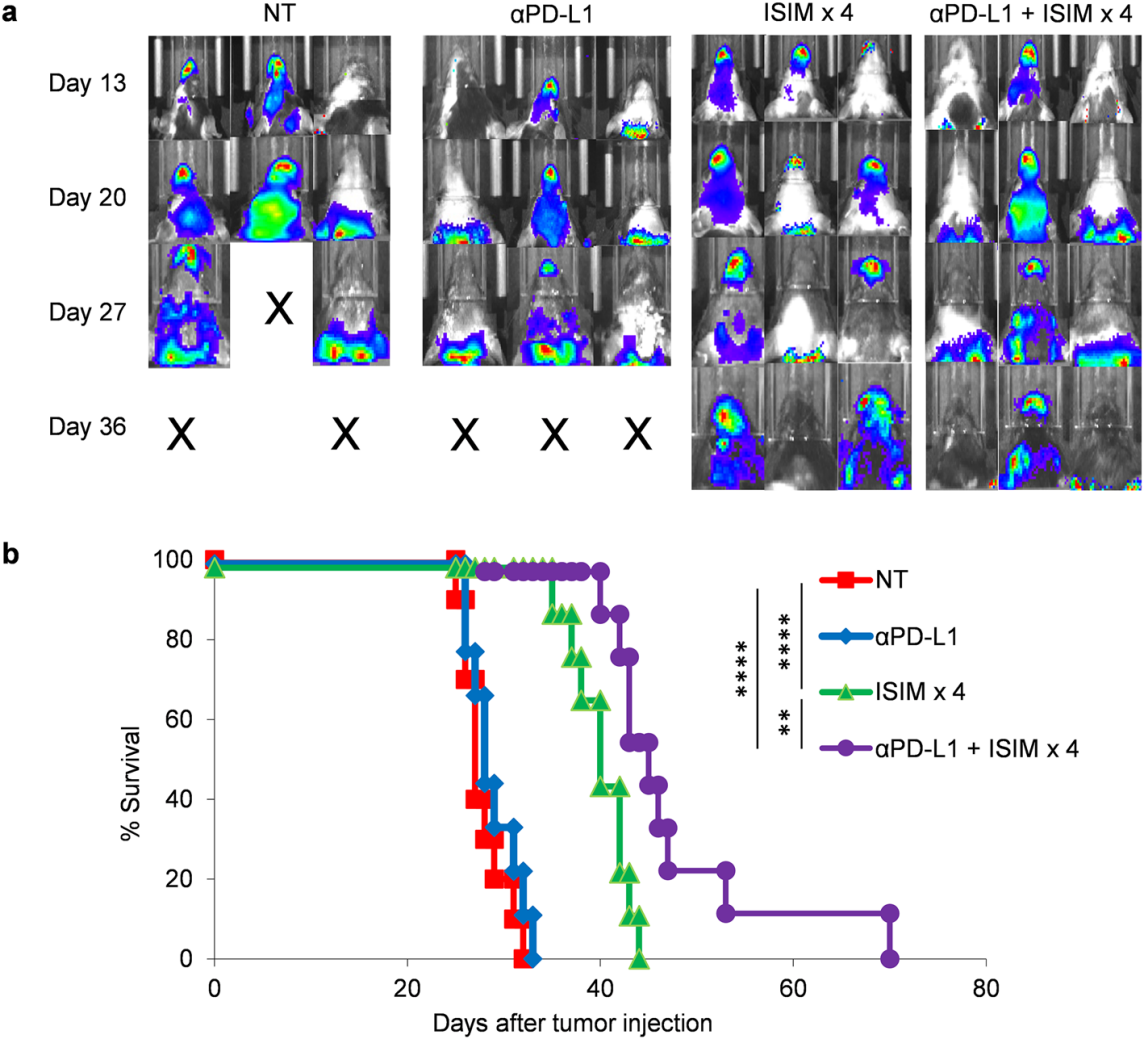
Serial ISIM treatment of mammary tumors and anti-PD-L1 therapy exhibit synergistic antitumor efficacy and improves survival in mice with established brain metastases. (**a**,**b**) AT-3 and AT-3-luc tumor cells were inoculated into the fourth mammary gland and left ventricle, respectively. Orthotopic mammary tumors were treated with PBS (NT) or ISIM × 4 with anti-PD-L1 antibody (αPD-L1) or isotype Ab every third days for 6 times starting from the day radiotherapy performed. Representative bioluminescent imaging of AT-3-luc brain metastases (**a**) and survival curves (**b**) in mice receiving different treatment to mammary AT-3 tumors as indicated (n = 8–10 per group). **P < 0.01, ****P < 0.0001 log-rank (Mantel-Cox) test (**b**). NT *vs.* ISIMx4, hazard ratio (HR) 4.628, 95% confidential interval (CI) 1.482–14.44; NT *vs.* αPD-L1 + ISIMx4, HR 4.628 95% CI 1.482–14.44; ISIMx4 *vs.* αPD-L1 + ISIM × 4 HR 3.053 95% CI 1.045–8.914. Data shown are representative of two independent experiments.

We have recently reported that ISIM treatment with Flt3L, radiotherapy and TLR3/CD40 agonists could reshape intratumoral T cell type, density, and repertoires, convert poorly T cell-inflamed tumors, and overcome primary and acquired resistance to anti-PD-L1 therapy^29^. However, it remained unknown whether anti-PD-L1 therapy potentiates the abscopal effect of serial ISIM treatment against brain metastases. To this end, we treated mice bearing established orthotopic mammary tumors and brain metastases with serial ISIM and anti-PD-L1 therapy. Treatment with anti-PD-L1 therapy alone did not improve control of brain metastases or survival compared to untreated mice (Fig. 4a,b). In contrast, serial ISIM treatment significantly improved survival and further synergized with anti-PD-L1 therapy. Collectively, these findings suggest that in situ induction and activation of cDC1s in the mammary tumors generates potent systemic antitumor immunity, delays the formation of established distant brain metastases, overcomes resistance to anti-PD-L1 therapy, renders them responsive to anti-PD-L1 therapy and improves survival.

## Discussion

Breast cancer is considered immunologically quiescent, representing a challenge for immunotherapy. Although TNBC has a higher rate of PD-L1 expression associated with the presence of tumor-infiltrating lymphocytes, and a higher degree of mutational burden compared with other subtypes of breast cancer^4–8^, the overall response rate to PD-1/PD-L1 blockade therapy is still low^13^. Due to limited treatment options and a lack of proven effective targeted therapies, there is a critical need for the development of new therapeutic approaches for patients with TNBC. The work described herein demonstrates that in situ induction and activation of cDC1s in poorly T cell-inflamed tumors facilitates the infiltration of CD8^+^ T cells in distant non-irradiated CNS lesions, delays the progression of established TNBC brain metastases, renders them responsive to anti-PD-L1 therapy and improves survival.

Our approach to improve anti-PD-1/PD-L1 blockade therapy focuses on converting non- or poorly T cell-inflamed tumors to T-cell inflamed ones. Our findings of increased CD8^+^ T cells and delayed progression of established TNBC brain metastases by ISIM treatment of mammary tumors are in line with our recent data in mouse models bearing bilateral mammary tumors^29^, and further demonstrated that this can occur in metastatic lesions that cross the blood–brain-barrier. Evidence suggests that T cells within the TME of the CNS are responsible for therapeutic efficacy of anti-PD-1 therapy^38^. High density of TILs is associated with better prognosis not only in various solid malignant tumors but also in brain metastases^39^, suggesting that ISIM with or without anti-PD-1/PD-L1 therapy may represent a novel approach that could potentially improve clinical outcome in at least some patients with TNBC brain metastases.

Local irradiation increases the levels of tumor-residing DCs, enhances the mobilization of these cells into the TdLN, augments their maturation, and increases their ability to cross-present antigens (Ags) to prime T cells^40–45^. However, this process is often hindered by the immunosuppressive TME, and the abscopal effect is only rarely seen in patients even in the presence of immune checkpoint inhibitors^46–51^. Our data indicate that in situ induction and activation of cDC1s augments immunogenicity of radiotherapy against untreated poorly T cell-inflamed tumors and generates abscopal effects in brain metastases. Optimal dose and fractionation of radiotherapy to the primary tumor after induction of cDC1s; however, remain unknown. Too small of a dose might not cause significant immunogenic cell death and the release of tumor Ags, but too much of a dose or too many cycles of irradiation might alter the viability of Flt3L-induced cDC1s, or negatively impact their ability to traffic to the TdLN and/or capacity to cross-present TAAs. Therefore, more work is warranted to determine the optimal radiotherapy regimen to maximize the engagement of cDC1s to enhance ISIM-induced abscopal effects. ISIM has some features that might be applicable for the treatment of unresectable and metastatic TNBC. First, intralesional therapy allows for achieving higher concentrations of immunomodulatory agents in the TME, while minimizing systemic toxicities. Second, this local combinatorial treatment may allow for the use of concurrent systemic therapy, such as chemotherapy plus anti-PD-1/PD-L1 therapy, and potentiate their antitumor efficacy because of ISIM-mediated increases in CD8^+^ T cell infiltration in mammary tumors, but more importantly distant metastatic lesions. Third, ISIM could cause robust regression of distant untreated tumors^29^, which might be important for patients with visceral metastases that are extremely challenging to control or eradicate. Several potential limitations exist in the ISIM treatment in clinical setting. First, the primary tumors must be palpable or accessible under image guidance for repetitive injections. Second, antitumor efficacy of ISIM depends on de novo adaptive T-cell immune responses elicited at secondary lymphoid organs^29^. Therefore, therapeutic efficacy of ISIM for patients with history of regional lymph node surgery remains to be determined. Third, although rapid regression of the treated and distant untreated tumors was observed in preclinical models^29^, it remains unclear whether this is the case against brain metastases when immediate intervention is required. Current therapy for brain metastases of breast cancer involves radiotherapy and surgery. Compelling evidence has shown that systemic Flt3L injections could induce tumor-residing cDC1s^24,25,27,28^ and synergize with radiotherapy and systemic anti-CD40 Ab^27^. Therefore, it is possible that irradiating brain metastases may have additive or synergistic antitumor efficacy with ISIM treatment of mammary tumors with or without anti-PD-L1 therapy. Although the scope of our studies is limited to assess the abscopal effect of ISIM, future investigations are necessary to evaluate the potential synergy of ISIM and radiotherapy to brain metastases.

Approaches that take advantage of the induction and activation of tumor-residing cDC1s has gained considerable attention recently^17,18,24–29^. In situ vaccination with Flt3L, radiotherapy and Poly-ICLC mediates regression of distant lesions in patients with lymphoma^26^, and a clinical trial with anti-PD-1 therapy is under way for patients including breast cancer (NCT03789097). A clinical trial involving subcutaneous injections of Flt3L and radiotherapy in combination with anti-PD-1 therapy for patients with breast cancer is ongoing (NCT03804944). Systemic administration of Flt3L and agonistic anti-CD40 Ab are also being tested for patients with pancreatic cancer (NCT04536077). Although we have shown delayed progression of established brain metastases in response to ISIM treatment of mammary tumors, the safety and feasibility of ISIM still needs to be evaluated in a clinical setting (NCT04616248) before its use in patients with synchronous mammary tumors with and brain metastatic lesions.

In summary, we evaluated the systemic antitumor efficacy against brain metastases by combinatorial intralesional multimodal therapy using a preclinical model of poorly T cell-inflamed TNBC. We also demonstrated that therapeutic responses could be further potentiated by the addition of anti-PD-L1 therapy. Collectively, these data have important implications for the design and use of therapeutic strategies that activate and engage cDC1-CD8^+^ T cell interactions to target distant metastases, thus providing the rationale for ISIM-based immunotherapy in patients with metastatic TNBC.

## Supplementary Information

Supplementary Information.

## Data Availability

All data generated and analyzed are available from the corresponding author upon reasonable request.
