# Supplementary material for: Local, multimodal intralesional therapy renders distant brain metastases susceptible to PD-L1 blockade in a preclinical model of triple-negative breast cancer
Source: Sci Rep. 2021 Nov 9;11:21992. doi: 10.1038/s41598-021-01455-4 (PMC8578367; doi:10.1038/s41598-021-01455-4)
Supplement: Supplementary file 1 — Supplementary Information. [file 41598_2021_1455_MOESM1_ESM.pdf]

Local, multimodal intralesional therapy renders distant brain metastases susceptible to PD-L1 blockade in a preclinical model of triple-negative breast cancer

Yokoi et al.

Supplementary Information

a

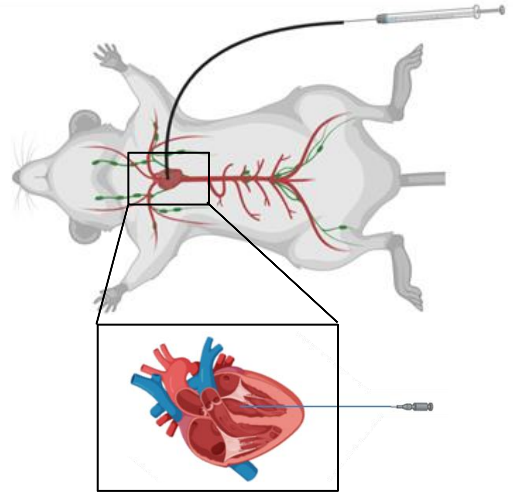

b

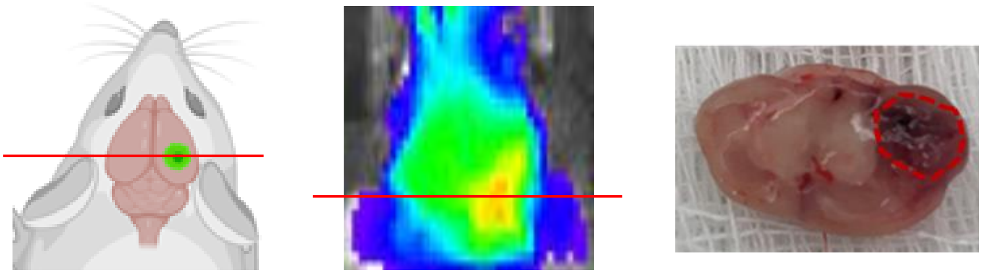

Supplementary Fig. 1 Establishment of brain metastases.

a, Scheme of intracardiac injection of tumor cells for establishment of brain metastases. AT-3-luc (1×10^6) tumor cells were injected through the fourth intra-costal space into the left ventricle under anesthesia with isoflurane

b, Scheme, (*in vivo* imaging system) IVIS imaging, and gross appearance of mouse brain showing the metastatic AT-3-luc tumors. Illustrations were created with BioRender.com.
